# IL-6 Amplifies TLR Mediated Cytokine and Chemokine Production: Implications for the Pathogenesis of Rheumatic Inflammatory Diseases

**DOI:** 10.1371/journal.pone.0107886

**Published:** 2014-10-01

**Authors:** Ivan Caiello, Gaetana Minnone, Dirk Holzinger, Thomas Vogl, Giusi Prencipe, Antonio Manzo, Fabrizio De Benedetti, Raffaele Strippoli

**Affiliations:** 1 Division of Rheumatology, Bambino Gesù Children’s Hospital, Rome, Italy; 2 Department of Paediatric Rheumatology and Immunology, University Children’s Hospital Muenster, Muenster, Germany; 3 Institute of Immunology, University Hospital Muenster, Muenster, Germany; 4 Rheumatology and Translational Immunology Research Laboratories (LaRIT), Division of Rheumatology, IRCCS Policlinico S. Matteo Foundation/University of Pavia, Pavia, Italy; 5 Department of Cellular Biotechnologies and Haematology, Sapienza University of Rome, Rome, Italy; Harvard Medical School, United States of America

## Abstract

The role of Interleukin(IL)-6 in the pathogenesis of joint and systemic inflammation in rheumatoid arthritis (RA) and systemic juvenile idiopathic arthritis (s-JIA) has been clearly demonstrated. However, the mechanisms by which IL-6 contributes to the pathogenesis are not completely understood. This study investigates whether IL-6 affects, alone or upon toll like receptor (TLR) ligand stimulation, the production of inflammatory cytokines and chemokines in human peripheral blood mononuclear cells (PBMCs), synovial fluid mononuclear cells from JIA patients (SFMCs) and fibroblast-like synoviocytes from rheumatoid arthritis patients (RA synoviocytes) and signalling pathways involved. PBMCs were pre-treated with IL-6 and soluble IL-6 Receptor (sIL-6R). SFMCs and RA synoviocytes were pre-treated with IL-6/sIL-6R or sIL-6R, alone or in combination with Tocilizumab (TCZ). Cells were stimulated with LPS, S100A8-9, poly(I-C), CpG, Pam2CSK4, MDP, IL-1β. Treatment of PBMCs with IL-6 induced production of TNF-α, CXCL8, and CCL2, but not IL-1β. Addition of IL-6 to the same cells after stimulation with poly(I-C), CpG, Pam2CSK4, and MDP induced a significant increase in IL-1β and CXCL8, but not TNF-α production compared with TLR ligands alone. This enhanced production of IL-1β and CXCL8 paralleled increased p65 NF-κB activation. In contrast, addition of IL-6 to PBMCs stimulated with LPS or S100A8-9 (TLR-4 ligands) led to reduction of IL-1β, TNF-α and CXCL8 with reduced p65 NF-κB activation. IL-6/IL-1β co-stimulation increased CXCL8, CCL2 and IL-6 production. Addition of IL-6 to SFMCs stimulated with LPS or S100A8 increased CXCL8, CCL2 and IL-1β production. Treatment of RA synoviocytes with sIL-6R increased IL-6, CXCL8 and CCL2 production, with increased STAT3 and p65 NF-κB phosphorylation. Our results suggest that IL-6 amplifies TLR-induced inflammatory response. This effect may be relevant in the presence of high IL-6 and sIL-6R levels, such as in arthritic joints in the context of stimulation by endogenous TLR ligands.

## Introduction

Interleukin-6 (IL-6) is a pleiotropic cytokine with multiple functions in different pathophysiologic systems [1], [2]. Data from animal models have shown that IL-6 plays a non-redundant role in several pathophysiological events, such as fever, liver acute-phase response, and in the transition from acute to chronic inflammation [3]. The recent introduction of tocilizumab (TCZ), an IL-6 receptor blocker, in the treatment of rheumatoid arthritis (RA) and systemic juvenile idiopathic arthritis (s-JIA), clearly demonstrated a major role of this cytokine in the pathogenesis of joint and systemic inflammation [4], [5]. However, the cellular and molecular mechanisms by which high levels of IL-6, which are present in blood and synovial fluids contribute to the pathogenesis are not completely understood.

In recent years, many efforts in understanding the pathogenic role of IL-6 in arthritis have been focused on the effect of IL-6 on adaptive immunity. Indeed, IL-6 has a role in the differentiation of B lymphocyte into auto-antibody producing plasma cells, which participate in the pathogenesis of arthritis through the formation of immune complexes (IC) [6]. Moreover, high levels of IL-6 may cause a Th17/Treg cell imbalance during RA, which is corrected upon treatment with TCZ [7], [8]. Accordingly, IL-6 mediated B and T lymphocyte dysregulation may lead to abnormal production of inflammatory cytokines, chemokines and metalloproteases by both leukocytes and cells of the synovial stroma, leading to synovial tissue degradation and bone erosions.

However, little is known about possible direct effects of IL-6 on innate immune mechanisms. Toll like receptors (TLRs) are a family of trans-membrane glycoproteins with conserved extracellular domains and a cytoplasmic signaling domain homologous to that of IL-1R, called the Toll/IL-1R domain. They bind a wide array of bacterial and viral peptides [9]. Signals from the various TLR ligands generally converge to activate the mitogen-activated protein kinases (MAPKs), p65 nuclear-factor kappa B (p65 NF-κB), and interferon regulatory factor 3 (IRF-3)/IRF-7 pathways, which mediate inflammatory cytokine and type I interferon secretion, thus controlling the response to pathogens [9]. TLR response may be relevant for the pathogenesis of rheumatic diseases also in the absence of infections. Indeed, a number of endogenous TLR ligands, especially for TLR2 and TLR4, has recently emerged as potential players in chronic inflammation [10]. For example, TLR4 ligands belonging to the S100 family may play a role in pathogenesis of s-JIA, and immunocomplexes (IC) including citrullinated proteins have been involved in the pathogenesis of RA [11]–[13].

We have previously shown that IL-6 directly enhances TRL ligand-induced response [14] both in vitro and ex vivo. IL-6 transgenic (IL-6TG) mice undergo an exaggerated inflammatory response when treated with TLR ligands and peritoneal macrophages from these mice produce increased levels of IL-6 and IL-1β when stimulated *ex vivo* with TLR ligands [14].

The aim of this study was to evaluate whether IL-6 directly affected inflammatory cytokine and chemokine production upon TLR stimulation with various TLR ligands in human mononuclear cells and RA synoviocytes.

## Methods

### Antibodies and chemicals

Polyclonal antibodies against phospho-STAT3 (Tyr705) phospho-NFκB (Ser536) were from Cell Signaling (Cell Signaling Technology, Danvers, MA); monoclonal antibodies against tubulin were from Sigma (Saint Louis, MO). Recombinant human IL-6 ad soluble IL-6 receptor (sIL-6R) were from R&D Systems (Minneapolis, MN). LPS was from Sigma Aldrich, (St Louis, MO) CpG-DNA, Pam3CSK4, Muramyl dipeptide (MDP) and Poly(IC) were from Invivogen (San Diego, CA); S100A8 and S100A9 were from Vogl lab. TCZ (RoActemra) was from Roche, Milan, Italy.

### Isolation and culture of peripheral blood mononuclear cells (PBMCs) synovial fluid mononuclear cells (SFMCs) and synovial fibroblasts form RA patients

Peripheral blood mononuclear cells (PBMCs) were derived from healthy donors by centrifugation on a LSF-Lymphocyte Separation Ficoll (LiStarFish). For ELISA studies, cells were put at 1×10^6^/well in 24 wells plate in DMEM supplemented with 10% fetal calf serum (FCS) (HyClone). For western blot assays, cells were starved in DMEM supplemented with 0.5% FCSfor 2 hours before stimulation. In some experiments, cells were left to adhere on plastic for 3 hours in DMEM supplemented with 10% FCS.

Synovial fluid mononuclear cells from 10 oligoarticular JIA patients (SFMCs) were isolated by Ficoll density centrifugation. 1×10^6^ cells/well were put in 24 wells plate and were left to adhere on plastic for 3 hours in DMEM supplemented with 10% FCS. Cells were then washed and treated with different stimuli. Due to limited amount of supernatants obtained by culture of SFMCs, it was not possible to measure all cytokines in the supernatants of the SFMCs obtained by each patient.

Synoviocytes were obtained from 5 RA patients undergoing ultrasound-guided synovial biopsy. [15]. Cells isolated from synovial tissue by enzymatic digestion with collagenase and were cultured in DMEM supplemented with 10% FCS. For ELISA assays, cells were grown to confluence in a p24 plate (approximately 50.000 cells per well). For western blot assays, cells were starved at 0.5% FCS for two hours before stimulation.

The part of the study involving JIA patients (SFMCs) was approved by the Ethical Committee of the Bambino Gesù Children’s Hospital Rome, Italy, and written consent was obtained from the parents of each child. The part of the study involving RA patients (synoviocytes) was approved by the Ethical Committee of the IRCCS Policlinico San Matteo Foundation, Pavia, Italy, and written consent was obtained from all patients.

### Reverse transcription–polymerase chain reaction (PCR)

Total RNA was extracted using TRIzol (Invitrogen), and cDNA was obtained using the Superscript Vilo kit (Invitrogen). The following gene expression assay was used in the real-time PCR assays: human TLR4 (Hs00152939_m1). Gene expression data were normalized using GAPDH (Hs99999905_m1) as an endogenous control and analyzed using the 2^ΔΔct^ method.

### Western blotting

Cells were lysed and Western blots were performed as in [14].

### Cytokine measurement

Human IL-6, IL-1β, TNF-α, CXCL8, CCL2 ELISA kits were from R&D Systems, Minneapolis, MN. All cytokines were quantified according to the manufacturers’ instructions.

### Statistical analysis

Data are expressed as the mean ± SEM. Statistical analysis was performed by one-way analysis of variance, followed by Student’s unpaired *t*-test or the Mann-Whitney U test. *P* values less than 0.05 were considered significant.

## Results

### IL-6 induces production of inflammatory chemokines by peripheral blood mononuclear cells

We first analyzed the effect of IL-6 on inflammatory cytokine and chemokine production by peripheral blood mononuclear cells (PBMCs). In all experiments, we used IL-6 in combination with the sIL-6R (IL-6/sIL-6R) in order to obtain a response also in cells expressing gp130, but not membrane IL-6 receptor (IL-6R) [2]. We used IL-6/sIL-6R at concentrations comparable con that observed in biological fluids (blood, synovial fluid) in physiopathological conditions [16].

**Figure 1:**
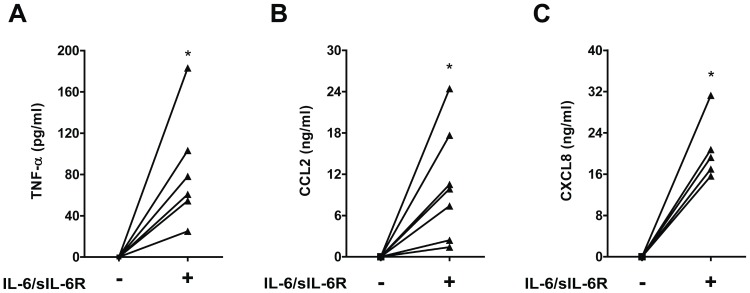
TNF-α, CCL2 and CXCL8 production in human PBMCs exposed to IL-6. Human PBMCs were exposed to IL-6 (10 ng/ml) in combination with sIL-6R (125 ng/ml) (IL-6/sIL-6R) for 18 hours. TNF-α, CCL2 and CXCL8 levels were measured by ELISA. *p<0.05 for values from IL-6/sIL-6R stimulated compared with not treated (NT) cells.

Stimulation with IL-6/sIL-6R for 18 hours induced production of TNF-α, CCL2 and CXCL8 (Figure 1). In contrast, no detectable levels of IL-1β protein were found (data not shown).

### IL-6 induces increased production of inflammatory cytokines and chemokines following TLR stimulation of mononuclear cells

**Figure 2:**
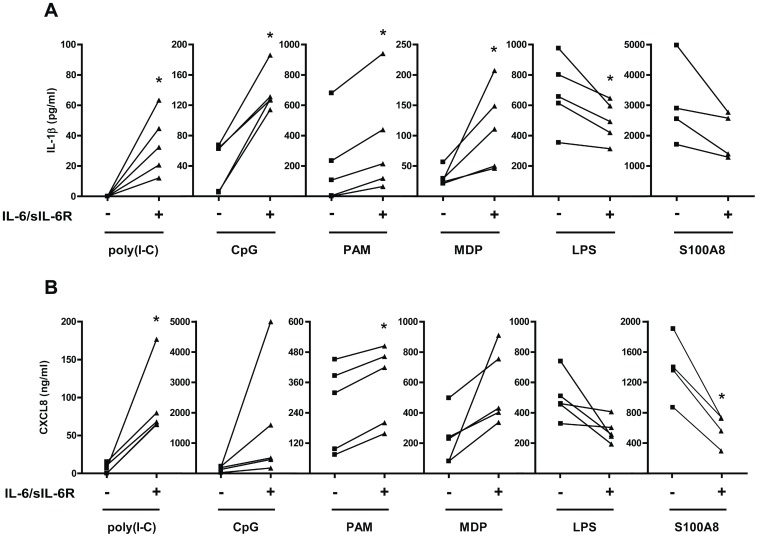
Exposure to IL-6 affects the production of inflammatory cytokines and chemokines in human PBMCs in response to TLR ligands. Human PBMCs were pre-exposed to IL-6/sIL-6R for 1 hour. Cells were then stimulated with poly(I-C) (20 µg/ml), CpG (5 µg/ml), PAM (200 ng/ml), MDP (10 µg/ml), LPS (10 ng/ml), S100A8 (5 µg/ml) for 18 hours. IL-1β (**A**) and CXCL8 (**B**) levels were measured by ELISA. *p<0.05 for values from IL-6/sIL-6R-stimulated compared with NT cells.

We then analysed the production of inflammatory cytokines and chemokines in response to TLR ligands in PBMCs in the presence or in the absence of pre-treatment with IL-6/sIL-6R. When IL-6/sIL-6R were added together with poly(I-C) (TLR-3 ligand), CpG (TLR-9 ligand), Pam2CSK4 (PAM) (TLR-2 ligand), or the NOD2 specific agonist MDP, we observed a marked increase in IL-1β production. IL-6 enhanced IL-1β production to a different extent when using different TLR ligands (Figure 2A). IL-6 induced ex novo IL-1β production upon poly(I-C) stimulation, a 6 fold increase upon MDP stimulation and a 1.7 fold increase when stimulating cells with PAM. In contrast, and similarly to what has been previously reported by others [17], when IL-6/sIL-6R were added together with LPS, a TLR4 ligand, we found a decrease in IL-1β production. The same result was obtained using S100A8, as well as S100A9, endogenous ligands of TLR4 (Figure 2A and Figure S1A). Also, CXCL8 production was increased following stimulation with poly(I-C), CpG, PAM, and MDP in the presence of IL-6/sIL-6R. In contrast, CXCL8 production induced by LPS, S100A8 and S100A9 was inhibited by the addition of IL-6/sIL-6R (Figure 2B and Figure S1B). In order to enrich for monocytes, we performed adherence on plastic before stimulation. Similarly to unseparated PBMCs, IL-1β and CXCL8 production was increased in the presence of IL-6/sIL-6R following stimulation with all TLR ligands analyzed, except LPS (Figure S2A–B).

In accordance with previously published results, TNF-α production induced by LPS was significantly reduced by the presence of IL-6/sIL-6R, whereas production by CpG, PAM, and MDP was not significantly affected (Figure S3).

### IL-6 potentiates IL-1β-induced inflammatory chemokine production

**Figure 3:**
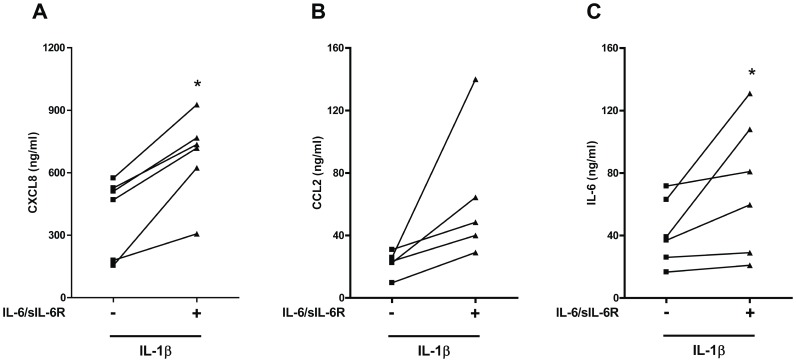
Exposure to IL-6 affects the production of inflammatory cytokines and chemokines in human PBMCs in response to IL-1β. Human PBMCs were pre-exposed to IL-6/sIL-6R for 1 hour. Cells were then stimulated with IL-1β (1 ng/ml) for 18 hours. CXCL8 (**A**), CCL2 (**B**), and IL-6 (**C**) levels were measured by ELISA. In (**C**), the levels of exogenous IL-6 were subtracted after the ELISA measurement. *p<0.05 for values from IL-6/sIL-6R-stimulated compared with NT cells.

Since the IL-1 receptor shares structural and functional similarities with TLRs, we analyzed the effect of IL-6 on IL-1β-induced inflammatory cytokine and chemokine production in PBMCs. IL- 6/sIL-6R enhanced IL-1β-induced production of CXCL8 (Figure 3A), CCL2 production (Figure 3B), and IL-6 (Figure 3C). On the other hand, IL-6/sIL-6R treatment did not significantly affect IL-1β-induced TNF-α production (Figure S4).

### IL-6 affects TLR signalling in PBMCs

**Figure 4:**
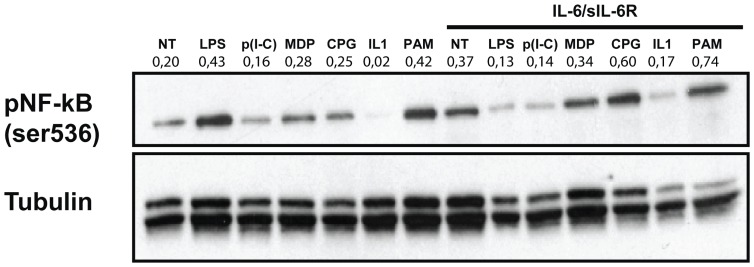
Increased p65 NF-κB activation in response to inflammatory stimuli in human PBMCs pre-exposed to IL-6/IL-6R. Western blots showing the expression of phospho–p65 NF-kB (Ser^536^) in total lysates from human PBMCs pre-exposed to IL-6 (10 ng/ml) in combination with sIL-6R (125 ng/ml) for 1 hour and stimulated with LPS, poly(I-C), MDP, CpG, IL-1β, PAM (used at the same concentrations as in Figure 2) for 90 minutes. Expression of α-tubulin was used as a loading control. Results of densitometric analysis are shown above each blot. Data are representative of 3 independent experiments.

We hypothesized that TLR and the IL-6 receptor cooperate in the induction of an increased pro inflammatory signalling in PBMCs. We focused on p65 NF-κB, where signals elicited by TLR and IL-6 may converge, as previously suggested by us and others [9], [14]. p65 NF-κB plays a major role in inflammatory cytokine and chemokine production upon TLR ligand stimulation and is involved in arthritis pathogenesis [9], [18]. We analyzed by western blot analysis Ser536 phosphorylation, which is linked to p65 NF-κB activity [19]. Differently from IκBα phosphorylation, Ser536 phosphorylation is not followed by p65 NF-κB ubiquitination and degradation and may better allow an analysis of the persistence of p65 NF-κB activation [20]. p65 NF-κB-DNA binding activity is often evaluated after cell stimulation for 1–2 h [20], [21]. Densitometry values were ratio-ed to that of tubulin. Treatment with IL-6/sIL-6R alone led to an increase in p65NF-κB phosphorylation induced by CpG, MDP, PAM and IL-1β (Figure 4). No changes were found in p65 NF-κB phosphorylation upon stimulation with poly(I-C). In contrast, IL-6/sIL-6R pretreatment led to a decrease in p65 NF-κB phosphorylation induced by LPS stimulation, consistently with the effect on cytokine production. Since different response to LPS upon exposure to IL-6 may be due to changes in receptor expression, we analyzed TLR4 expression by RT-PCR analysis, and we did not find clear changes (Figure S5). Thus, our results suggest that IL-6 may modify TLR mediated cytokine production through an effect on p65 NF-κB activation. Moreover, the reduced TLR4-mediated signaling upon exposure to IL-6 is not related to changes in TLR4 expression.

### IL-6 enhances inflammatory cytokine and chemokine production in SFMCs stimulated with TLR ligands

**Figure 5:**
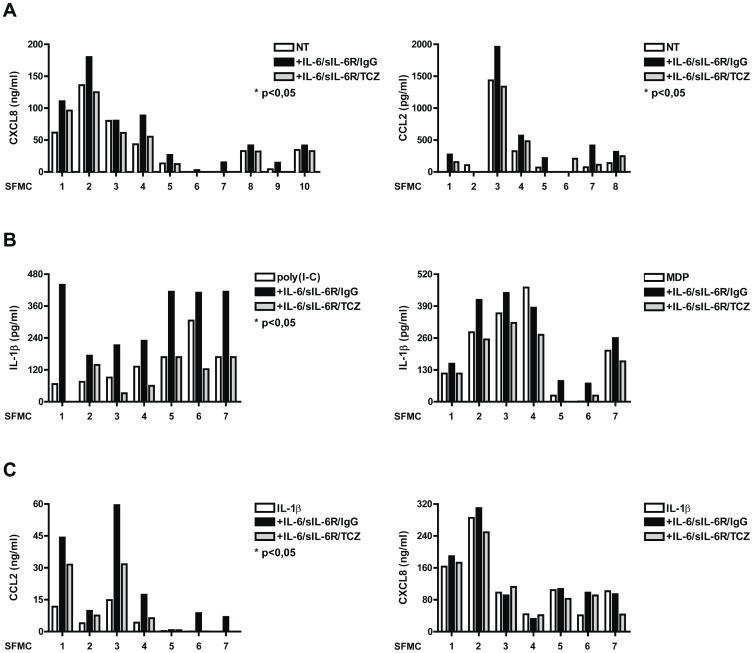
Exposure to IL-6 enhances cytokine and chemokine production in SFMC. SFMC were left to adhere on plastic for 3 hours in DMEM supplemented with 10% fetal calf serum (FCS). SFMC were pre-exposed to IL-6/sIL-6R in combination with IgG1 or with TCZ (50 µg/ml) for 1 hour. Cells were then left untreated (**A**) or stimulated with poly(I-C) and MDP (**B**), IL-1β (1ng/ml) (**C**). CXCL8, CCL2 and IL-1β levels were measured by ELISA. *p<0.05 for values from IL-6/sIL-6R-stimulated compared with NT cells.

In order to investigate the direct patho-physiological relevance of our finding, we analyzed whether IL-6 has a pro-inflammatory activity in mononuclear cells from synovial fluid (SFMCs) of JIA patients. IL-6 levels in synovial fluids of patients with JIA are markedly elevated [16]. We separated adherent cells in order to enrich for macrophages. In unstimulated conditions, these cells showed undetectable IL-1β production (data not shown), but produced high amounts of CXCL8 and CCL2 (Figure 5A). In these cells, IL-6/sIL-6R treatment increased CXCL8 and CCL2 production, which were reduced upon treatment with TCZ (Figure 5A). Similarly to PBMCs from human donors, IL-1β production in these cells was increased upon stimulation with poly(I-C) and MDP in the presence of IL-6/sIL-6R (Figure 5B). Accordingly, when stimulating these cells with IL-1β, treatment with IL-6/sIL-6R led to a further increase of CCL2, but not of CXCL8 production (Figure 5C).

**Figure 6:**
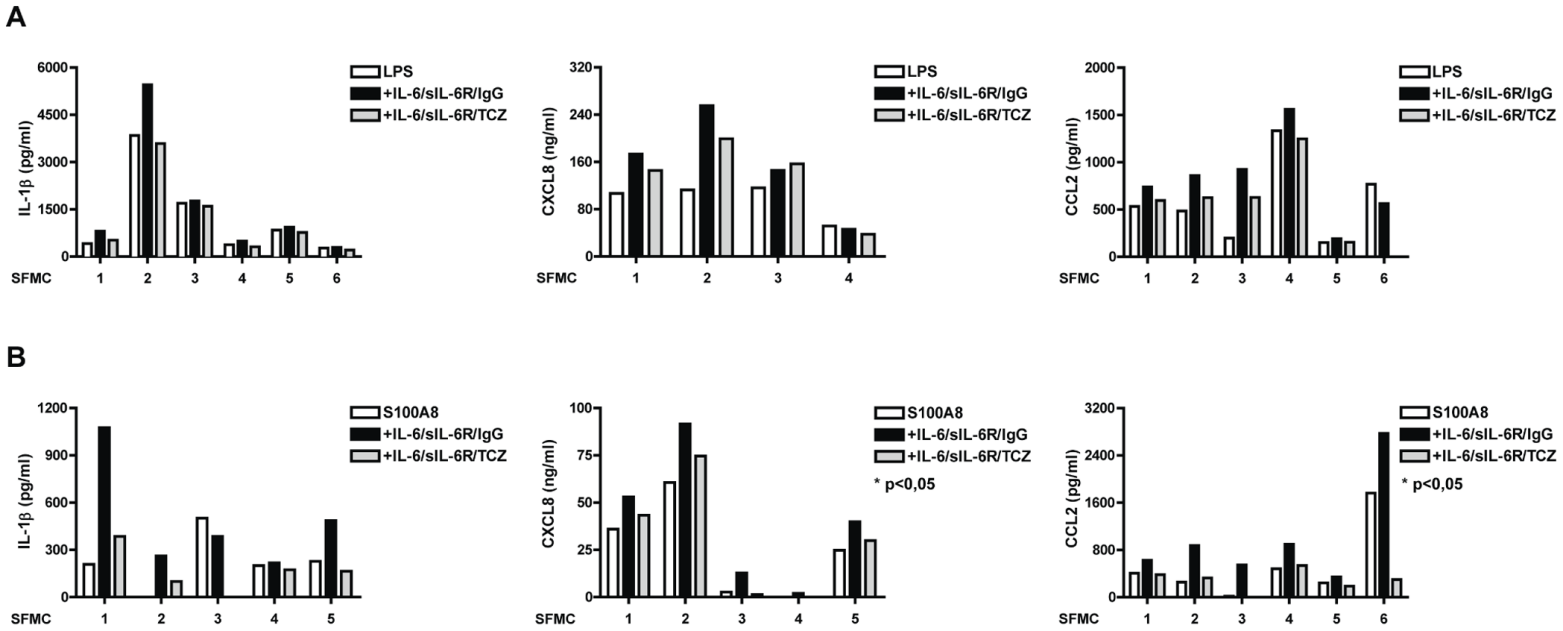
Exposure to IL-6 enhances cytokine and chemokine production in SFMC. SFMC were left to adhere on plastic for 3 hours in DMEM supplemented with 10% fetal calf serum (FCS). SFMC were pre-exposed to IL-6/sIL-6R in combination with IgG1 or with TCZ for 1 hour. Cells were then stimulated for 18 hours with LPS (10 ng/ml) (**A**), and S100A8 (5 µg/ml) (**B**). IL-1β, CXCL8 and CCL2 levels were measured by ELISA. *p<0.05 for values from IL-6/sIL-6R -stimulated compared with NT cells.

Interestingly, and differently from what observed with PBMCs when stimulating these cells with LPS or S100A8, as TLR4 ligands, pre-treatment with IL-6/sIL-6R led to increased CXCL8, CCL2, and IL-1β production (Figure 6A–B).

### IL-6 enhances inflammatory cytokine and chemokine production in RA synoviocytes

**Figure 7:**
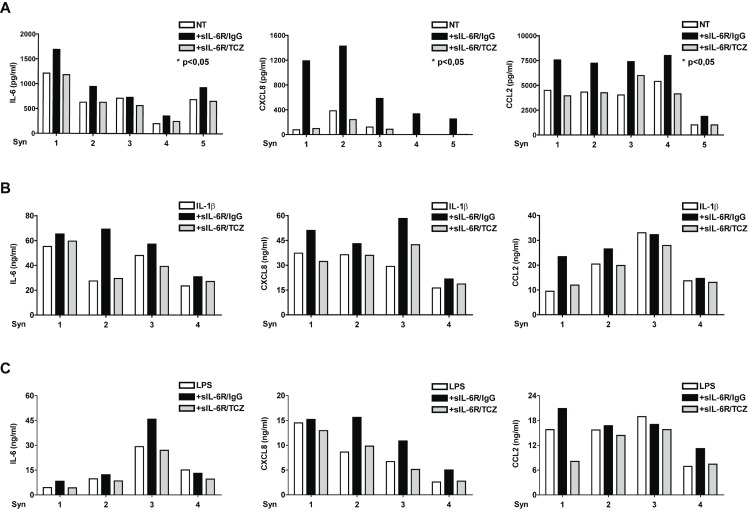
Exposure to sIL-6R enhances cytokine and chemokine production in RA synoviocytes. (**A**) Cells were treated with control IgG1, with sIL-6R in combination with IgG1 (sIL-6/IgG1) or with TCZ (sIL-6R/TCZ) for 18 hours. IL-6, CXCL8 and CCL2 levels were measured by ELISA. (**B–C**) RA synoviocytes were treated with control IgG1, with sIL-6/IgG1 or with sIL-6R/TCZ for 1 hour. Cells were then left untreated or stimulated with IL-1β (1 ng/ml) (**B**) or LPS (10 ng/ml) (**C**). CXCL8 and CCL2 levels were measured by ELISA. *p<0.05 for values from IL-6/sIL-6R -stimulated compared with NT cells.

**Figure 8:**
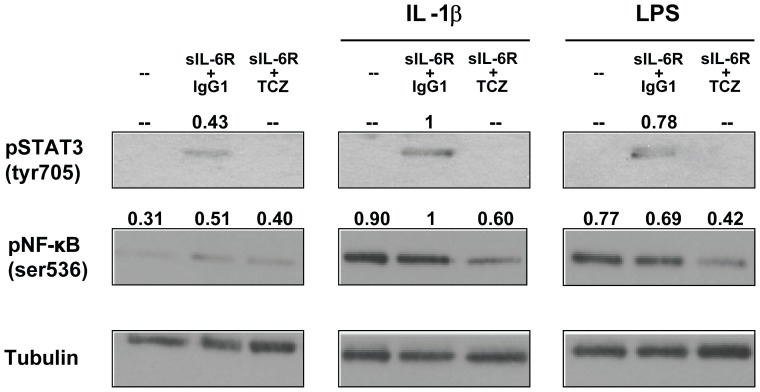
Western blots showing the expression of phospho–STAT3 (Tyr^705^), phospho–NF-kB (Ser^536^) in total lysates of RA synoviocytes pretreated as in Figure 7B for 1 hour and then stimulated with IL-1β (1 ng/ml) or LPS (10 ng/ml) for 90 minutes. Expression of α-tubulin was used as a loading control. Results of densitometric analysis are shown above each blot. Data are representative of 3 independent experiments.

We analyzed whether IL-6 exhibits its pro-inflammatory effect also in RA synoviocytes. Since these cells do not express the membrane IL-6R, they respond to IL-6 only in the presence of sIL-6R [22]. RA synoviocytes constitutively produced IL-6, which was increased by treatment with sIL-6R and inhibited by the addition of TCZ (Figure 7A). Treatment with sIL-6R alone led also to increased production of CXCL8 and CCL2 (Figure 7A). Moreover, addition of sIL-6R led to increased production of IL-6, CXCL8 and CCL2 following stimulation with IL-1β (Figure 7B) or LPS (Figure 7C). Analysis of lysates from RA synoviocytes showed that tyrosine phosphorylation of STAT3 was increased, confirming the activation of the IL-6 signalling pathway following addition of sIL-6R (Figure 8). Serine536-NF-κB phosphorylation was increased in the presence of sIL-6R alone and was markedly reduced by TCZ both in untreated cells and in cells stimulated with IL-1β and LPS. Overall, these results demonstrate that high levels of IL-6/sIL-6R, as those present in joints of patients with arthritis, may contribute to the amplification of the inflammatory response enhancing the production of IL-1β, CXCL8 and CCL2 by SFMCs and by RA synoviocytes.

## Discussion

Since its discovery, IL-6 has been described as a cytokine able to exert pro- as well as anti-inflammatory activities [17], [23]–[26]. Early studies unequivocally demonstrated that IL-6 inhibits the LPS-induced release of IL-1 and TNF-α in peripheral blood mononuclear cells [17], [27]. Our findings confirm and extend these results, obtained using LPS, showing that addition of IL-6 to PBMCs led to the inhibition of IL-1β and CXCL8 production not only when induced by LPS, but also when induced by S100A8-9, as an endogenous TLR4. In contrast, we found that, when PBMCs were stimulated with other stimuli such as poly(I-C), CpG and MDP, IL-6 cooperates with pro-inflammatory signals increasing IL-1β and CXCL8 production. To the best of our knowledge this has not been reported before. Overall, for all TLR ligands analysed, with the exception of TLR4-specific ligands, IL-6 enhanced IL-1β and CXCL8 production by PMBCs.

The inhibition observed with TLR4 ligands versus the stimulatory effect observed with other TLRs has no clear explanation in terms of different signaling pathways involved [9]. LPS-induced response is intense and has often been considered as a paradigm of TLR mediated response. However, it may not reflect the complexity of the activity of multiple TLR ligands. Indeed, TLR4 engagement leads to the induction of Myd88 dependent, as well as MyD88-independent pathways, involving the recruitment of TRIF adaptor protein upon activation. Dose response experiments with S100A8/9 and LPS ruled out that the inhibitory effect was related to the intensity of the response (data not shown). Also, expression of TLR4 was not affected by exposure to IL-6. Notably, we found that the addition of IL-6 led to reduced NF-κB activity upon LPS, but not other TLR ligand stimulation, suggesting profound, and yet to be clarified differences in the interactions between multiple signaling pathways.

We also focused on the IL-1β/IL-6 mutual regulation. IL-1β/IL-6 co-stimulation led to an increased production of CXCL8, CCL2 and IL-6. This potentiation between IL-1 and IL-6 may have a translational relevance. In patients with systemic JIA, recent results in clinical trials underscore a role for both IL-6 and IL-1β overproduction [4], [28]. It is well known that IL-1 induces IL-6 [17]; our data show synergy in the induction of pro-inflammatory chemokines. All together these data show multiple levels of interactions between IL-1β and IL-6 in vitro and led us to hypothesize an IL-6/IL-1β axis, rather than an IL-6/TNF-α axis, occurring during inflammation in the presence of TLR ligands. Clarifying IL-1/IL-6 mutual regulations and interactions in vivo, in patients with sJIA, may lead to a better understanding of disease pathogenesis and, possibly, of a way to predict response to treatments. A functional link between IL-1β and IL-6 is also suggested by the observation that patients with familial Mediterranean fever (FMF), an auto-inflammatory disease, were successfully treated with TCZ [29], [30].

In order to investigate whether the results observed with PBMCs were also reproduced in cells originating from inflammatory site, i.e. inflamed joints, we used adherent MCs obtained from synovial fluids of patients with JIA and synovial fibroblasts obtained from synovial tissue of patients with RA. Differently from PBMCs, SFMCs produce in steady state high levels of cytokines and chemokines. Treatment with IL-6 increased basal CXCL8 and CCL2 production and IL-1β production following poly(I-C) and MDP stimulation. More importantly, in contrast with PBMCs, we did not observe any inhibitory effect of IL-6 on CXCL8 and CCL2 production following TLR4 stimulation with both LPS and S100A8-9. Rather, a stimulatory effect was observed. In RA synoviocytes treatment with sIL-6R led to a *de novo* production of CXCL8 and to an increased production of IL-6 and CCL2. Noteworthy, addition of sIL-6R to IL-1β or LPS stimulated RA synoviocytes did not inhibit the production of IL-6, CXCL8 and CCL2. Similarly to what observed with SFMCs, a stimulatory effect was observed.

These data, with SFMCs and with RA synoviocytes suggest that the inhibitory effect of IL-6 on LPS induced cytokine production is cell and cell context specific. It is conceivable to speculate that cells who have been exposed to an inflammatory environment, such as SFMCs and RA synoviocytes, have abnormal regulation of their responses to *pro-*inflammatory stimuli. Our results point to a differential regulation of the p65 NF-κB pathway activation under these circumstances.

IL-6 has been demonstrated to enhance p65 NF-κB activation in intestinal epithelia, but no information to date has been provided about its activity in synoviocytes [31]. Our results led us to hypothesize that IL-6 and TLR induced intracellular signals may converge at the level of p65 NF-κB phosphorylation or upstream. Additional connections between the two pathways cannot be excluded. For instance, STAT-3 and p65 NF-κB have been shown to physically interact, enhancing CXCL8 transcription activity [32].

**Figure 9:**
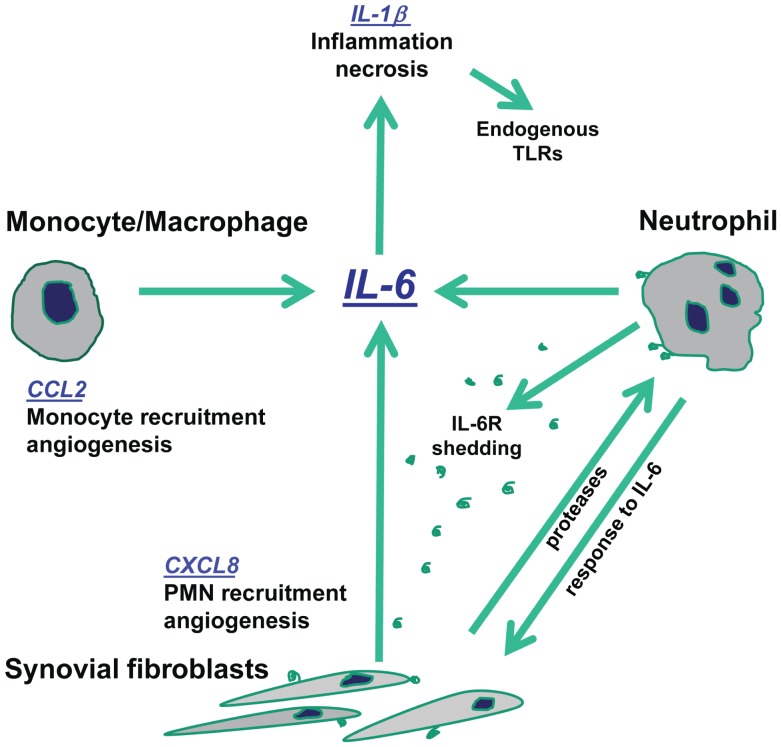
IL-6 amplifies the inflammatory response through a direct effect on stromal and innate immunity cells.

Our results in the context of the pathophysiological events occurring in inflamed joints suggest that the high levels of IL-6 may have a relevant role in amplifying inflammation. The interactions between stromal and innate immunity cells orchestrated by IL-6 are summarized in in Figure 9. IL-6 in the presence of its soluble receptor induces CXCL8 production by RA synoviocytes. CXCL8 induces polymorphonuclear neutrophil leukocyte (PMN) chemotaxis and activation. IL-6 is known to prolong the survival of these cells [33]. Proteases present in the synovial fluid will enhance the shedding of sIL-6R from the plasma membrane of PMNs, increasing IL-6 activity on RA synoviocytes [24]. IL-6 also plays a direct role in monocyte chemotaxis and in the differentiation of monocytes towards macrophages [33], [34]. Moreover, CXCL8 may contribute to neo-angiogenesis, which is a characteristic of inflamed synovia [35]. CCL2 also may enhance angiogenesis and contribute to chemotaxis of mononuclear cells [35]. Furthermore, our data point to endogenous TLR ligands released from necrotic synovial fluid cells cooperating with IL-6 in amplifying local inflammation [36].

Overall, our study shed light on the role of IL-6 in the pathogenesis of inflammatory arthritides. We demonstrated a direct effect of IL-6 in enhancing TLR ligand induced inflammatory cytokine and chemokine production. These IL-6 mediated effects support the presence of a positive loop between components of the innate immunity, such as monocyte/macrophages and neutrophils, and cells from the synovial stroma, such as RA synoviocytes.

## Supporting Information

Figure S1 **Effects of exposure to IL-6 on the production of IL1-β in human PBMCs in response to S110A9.** Human PBMCs were pre-exposed to IL-6/sIL-6R for 1 hour. Cells were then stimulated with S100A9 (5 µg/ml) for 18 hours. IL1-β (**A**) and CXCL8 levels (**B**) were measured by ELISA. *p<0.05 for values from IL-6/sIL-6R-stimulated compared with NT cells.
(TIF)

Figure S2 **Exposure to IL-6 affects the production of IL1-β and CXCL8 in adherent human PBMCs in response to TLR ligands.** Human PBMCs cells were left to adhere on plastic for 3 hours in DMEM supplemented with 10% fetal calf serum (FCS). Cells were pre-exposed to IL-6/sIL-6R for 1 hour. Cells were then stimulated with poly(I-C) (20 µg/ml), CpG (5 µg/ml), PAM (200 ng/ml), MDP (10 µg/ml), LPS (10 ng/ml) for 18 hours. IL-1β (**A**) and CXCL8 (**B**) levels were measured by ELISA. *p<0.05 for values from IL-6/sIL-6R-stimulated compared with NT cells.
(TIF)

Figure S3 **Effects of exposure to IL-6 on the production of TNF-α in human PBMCs in response to TLR ligands.** Human PBMCs were pre-exposed to IL-6/sIL-6R for 1 hour. Cells were then stimulated with CpG (5 µg/ml), PAM (200 ng/ml), poly(I-C) (20 µg/ml), MDP (10 µg/ml), LPS (10 ng/ml) for 18 hours. TNF-α levels were measured by ELISA. *p<0.05 for values from IL-6/sIL-6R-stimulated compared with NT cells.
(TIF)

Figure S4 **Effects of exposure to IL-6 on the production of TNF-α in human PBMCs in response to IL-1β.** Human PBMCs were pre-exposed to IL-6/sIL-6R for 1 hour. Cells were then stimulated with IL-1β (1 ng/ml) for 18 hours. CXCL8. TNF-α levels were measured by ELISA.
(TIF)

Figure S5 **Effects of exposure to IL-6 on the expression of TLR4 in human PBMCs in response to LPS.** Human PBMCs were pre-exposed to IL-6/sIL-6R for 1 hour. Cells were then stimulated with LPS (10 ng/ml) for 6 hours. Quantitative reverse transcription–polymerase chain reaction was performed on total RNA, with TLR4 mRNA normalized to GAPDH mRNA expression.
(TIF)
