# Supplementary figures and images for: IL-6 Amplifies TLR Mediated Cytokine and Chemokine Production: Implications for the Pathogenesis of Rheumatic Inflammatory Diseases
Source: PLoS One. 2014 Oct 1;9(10):e107886. doi: 10.1371/journal.pone.0107886 (PMC4182736; doi:10.1371/journal.pone.0107886)

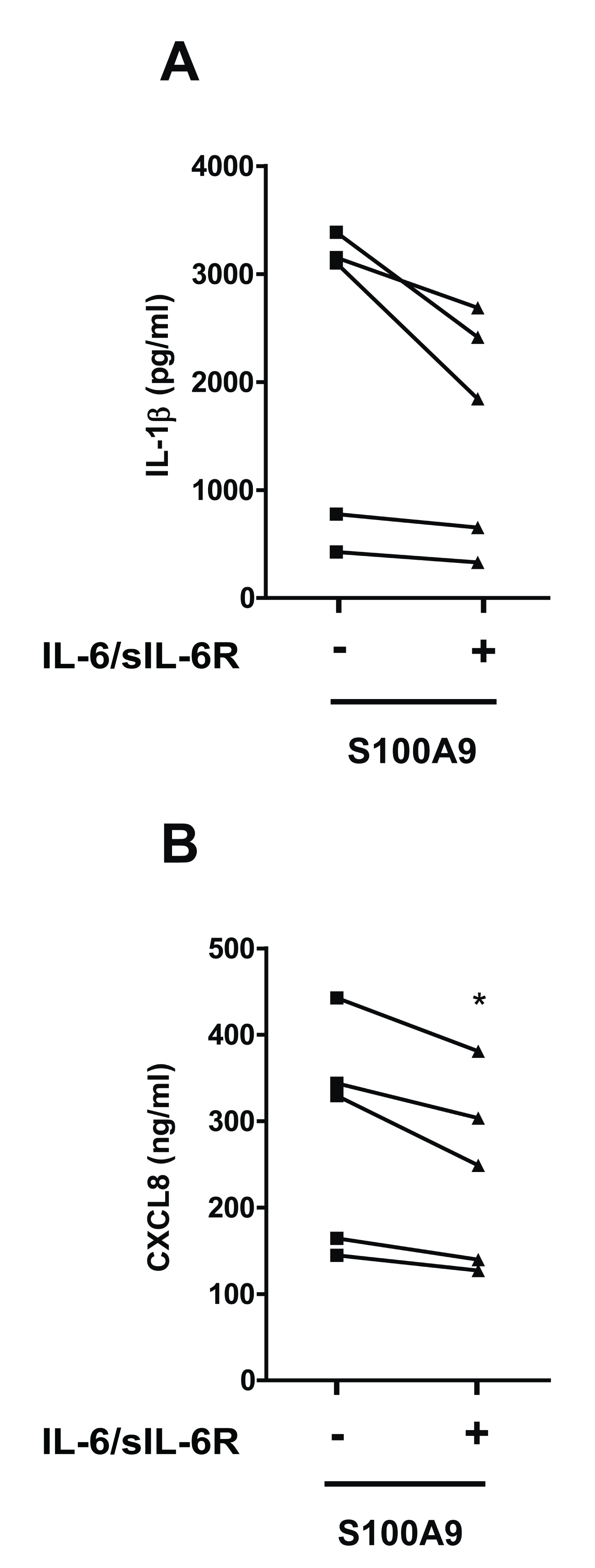

Supplement: Figure S1 — Effects of exposure to IL-6 on the production of IL1-β in human PBMCs in response to S110A9. Human PBMCs were pre-exposed to IL-6/sIL-6R for 1 hour. Cells were then stimulated with S100A9 (5 µg/ml) for 18 hours. IL1-β (A) and CXCL8 levels (B) were measured by ELISA. *p<0.05 for values from IL-6/sIL-6R-stimulated compared with NT cells. (TIF) [file pone.0107886.s001.tif]

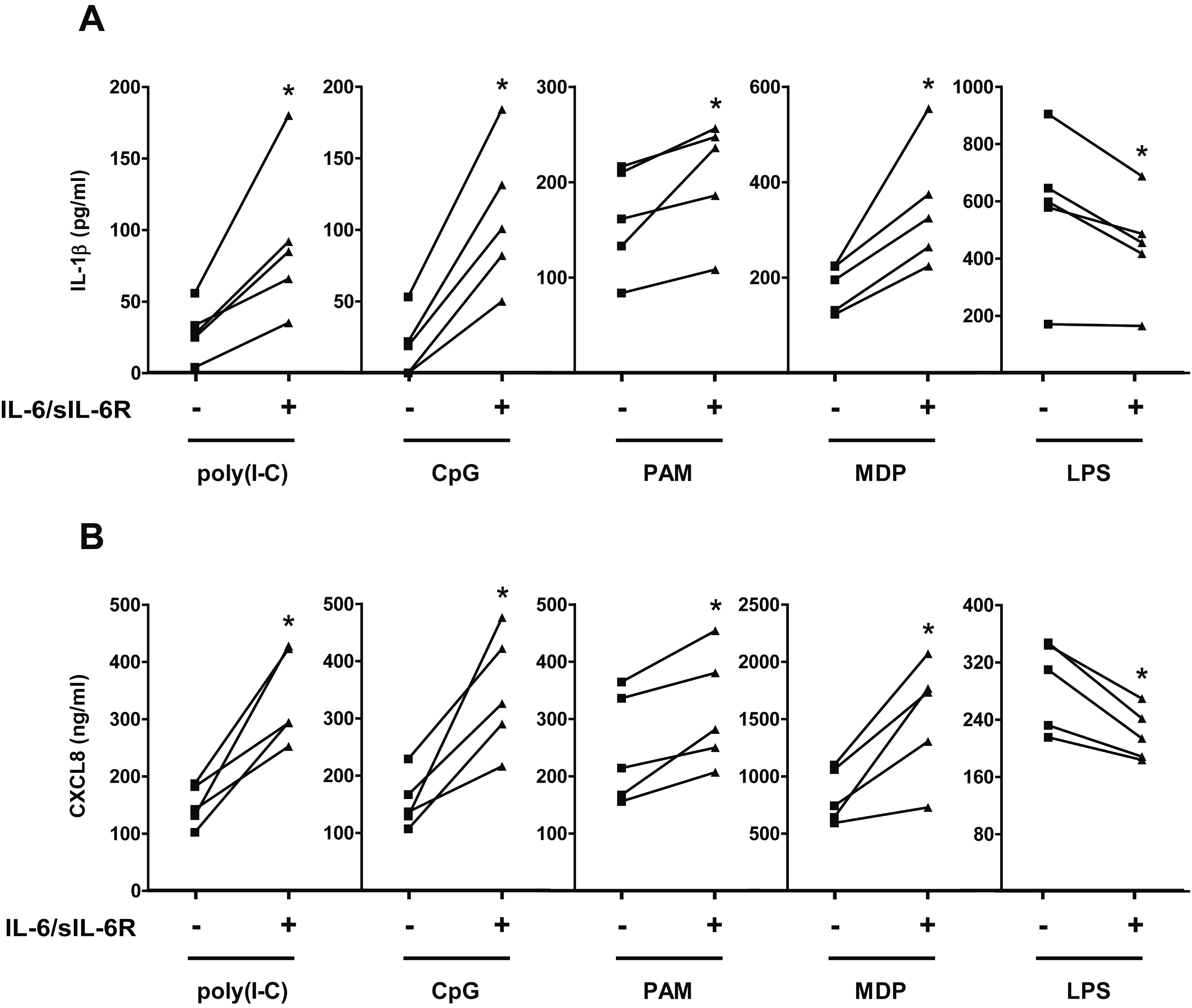

Supplement: Figure S2 — Exposure to IL-6 affects the production of IL1-β and CXCL8 in adherent human PBMCs in response to TLR ligands. Human PBMCs cells were left to adhere on plastic for 3 hours in DMEM supplemented with 10% fetal calf serum (FCS). Cells were pre-exposed to IL-6/sIL-6R for 1 hour. Cells were then stimulated with poly(I-C) (20 µg/ml), CpG (5 µg/ml), PAM (200 ng/ml), MDP (10 µg/ml), LPS (10 ng/ml) for 18 hours. IL-1β (A) and CXCL8 (B) levels were measured by ELISA. *p<0.05 for values from IL-6/sIL-6R-stimulated compared with NT cells. (TIF) [file pone.0107886.s002.tif]

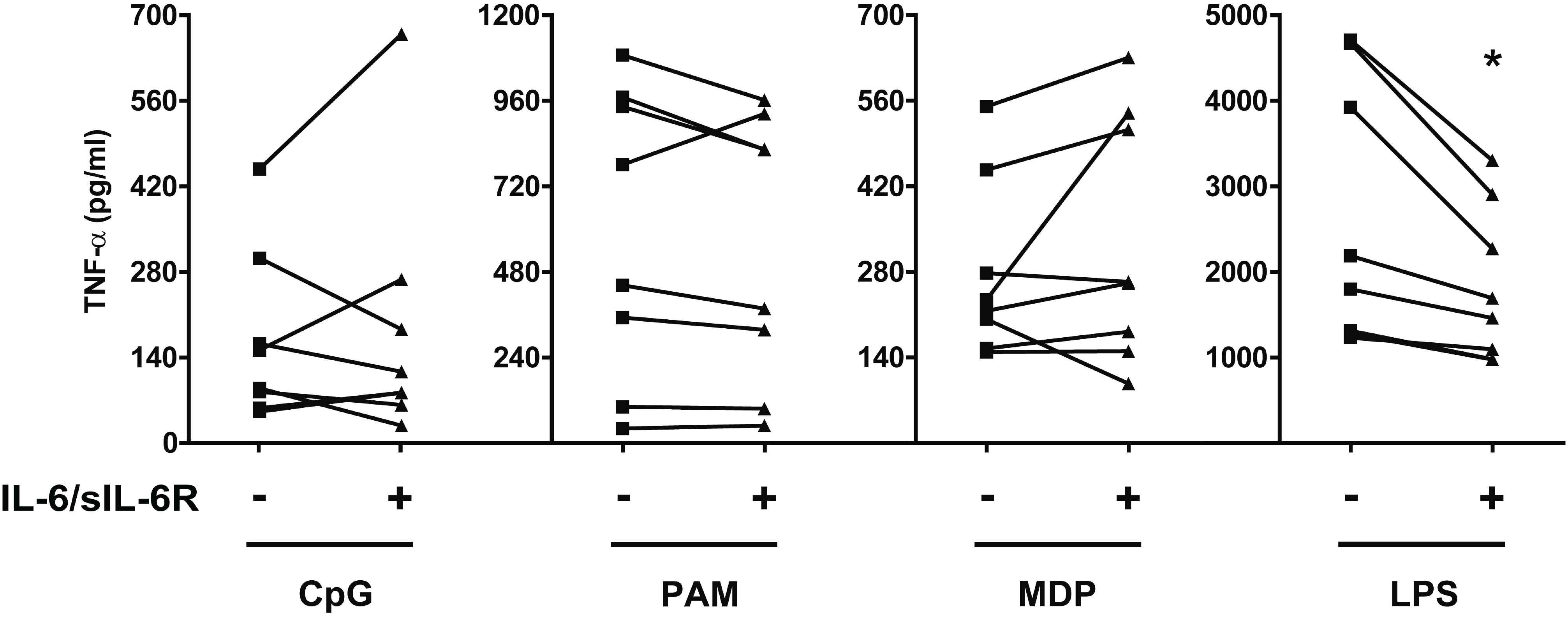

Supplement: Figure S3 — Effects of exposure to IL-6 on the production of TNF-α in human PBMCs in response to TLR ligands. Human PBMCs were pre-exposed to IL-6/sIL-6R for 1 hour. Cells were then stimulated with CpG (5 µg/ml), PAM (200 ng/ml), poly(I-C) (20 µg/ml), MDP (10 µg/ml), LPS (10 ng/ml) for 18 hours. TNF-α levels were measured by ELISA. *p<0.05 for values from IL-6/sIL-6R-stimulated compared with NT cells. (TIF) [file pone.0107886.s003.tif]

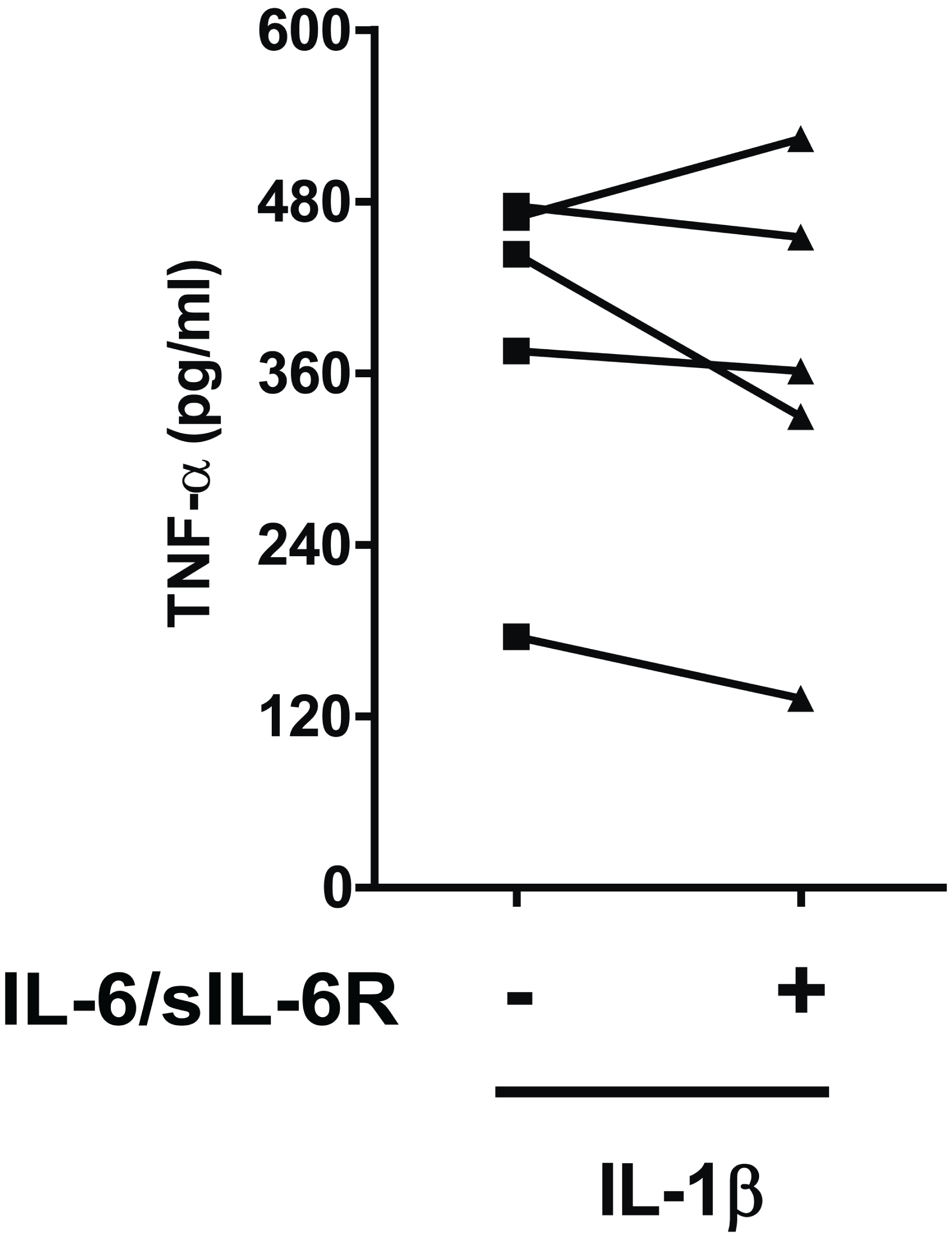

Supplement: Figure S4 — Effects of exposure to IL-6 on the production of TNF-α in human PBMCs in response to IL-1β. Human PBMCs were pre-exposed to IL-6/sIL-6R for 1 hour. Cells were then stimulated with IL-1β (1 ng/ml) for 18 hours. CXCL8. TNF-α levels were measured by ELISA. (TIF) [file pone.0107886.s004.tif]

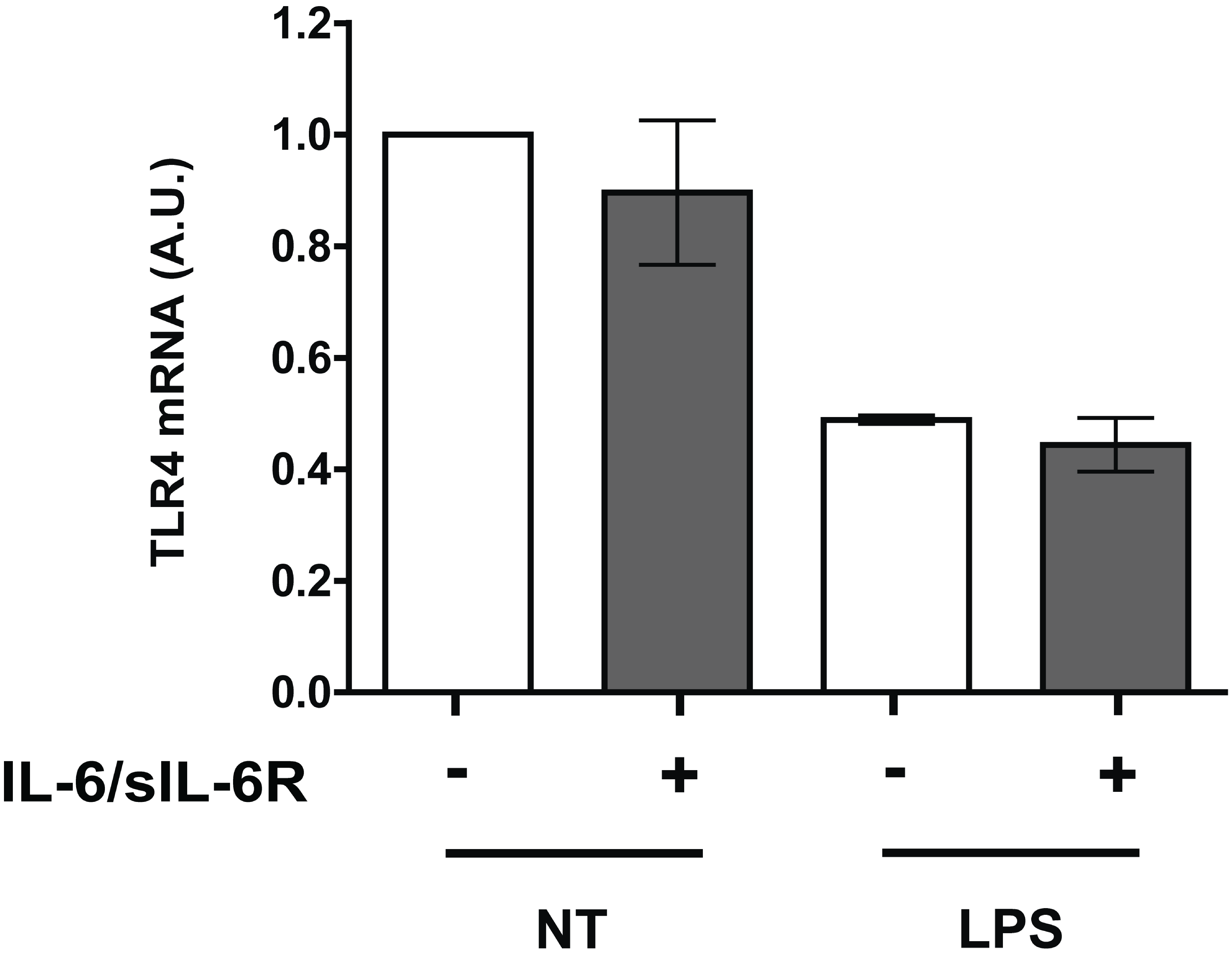

Supplement: Figure S5 — Effects of exposure to IL-6 on the expression of TLR4 in human PBMCs in response to LPS. Human PBMCs were pre-exposed to IL-6/sIL-6R for 1 hour. Cells were then stimulated with LPS (10 ng/ml) for 6 hours. Quantitative reverse transcription–polymerase chain reaction was performed on total RNA, with TLR4 mRNA normalized to GAPDH mRNA expression. (TIF) [file pone.0107886.s005.tif]
